# Functional analysis of distinct factors linked to the development of latent to active tuberculosis

**DOI:** 10.3389/fcimb.2026.1666138

**Published:** 2026-01-28

**Authors:** Karthikeyan Sundaram, Venkataraman Prabhu

**Affiliations:** 1 Department of Herbal Pharmacology and Environmental Sustainability, Chettinad Hospital and Research Institute, Chettinad Academy of Research and Education, Kelambakkam, Chennai, Tamilnadu, India; 2 Division of Medical Research, SRM Medical College Hospital and Research Centre, Kattankulathur, Chennai, Tamilnadu, India

**Keywords:** tuberculosis, active tuberculosis, latent tuberculosis, autophagy, immune response

## Abstract

Tuberculosis is an infectious disease spread through airborne droplet nuclei. *Mycobacterium tuberculosis* is the etiological agent of this infection. Mycobacteria can cause active tuberculosis or asymptomatic latent infection due to its complex biology and host immunological responses. The genes of mycobacteria can change alveolar macrophages and boost their resistance to autophagosome-lysosome fusion. However, only 5%–10% of infected individuals progress to the active form. In this context, multiple factors are associated with the progression of the disease. Thus, the review aims to analyze the essential factors linked to the progression from latent to active tuberculosis. The mycobacterium genome closely links these factors. Importantly, mycobacteria possess numerous genes to act as a self-defense mechanism against autophagosome-lysosome fusion. The *PE_PGRS* proteins play an essential role in this mechanism. This protein, when combined with *Rab1A*, helps activate *Rab1A* GTP, hence boosting *mTOR* and preventing autophagy. The presence of certain miRNAs, probably *miR-142-3p*, reduced the development of the phagosome in macrophages; *circRNA*-*0003528* helped change macrophages related to Mycobacterium by increasing CTLA4 and decreasing *miR-224-5p, miR-324-5p*, and *miR-488-5p*. Single-cell technologies like RNA sequencing can properly examine adaptive immune cell types in healthy people and patients, including CD4+, CD8+ T, and B cells. Deficiency of CD4+ T cells increases the risk of TB and can transform an infection into active tuberculosis. Therefore, research on autophagy-regulated genes and T-cell-mediated immune response, along with transcriptome analyses will determine the pathogenesis of tuberculosis, differentiate between active and latent TB, and facilitate the critical role of diagnostic biomarkers.

## 1 Introduction

The infectious disease tuberculosis (TB) is transmitted by air droplet nuclei, and the etiological agent of the infection is *Mycobacterium tuberculosis* (MTB), which is in the top 10 infectious agents that cause death. 10.8 million people (95% UI: 10.1–11.7 million) suffered from TB worldwide in 2023, and drug-resistant TB is a global threat, according to the WHO Global Tuberculosis Report 2024 (Global Tuberculosis Report, 2024; J et al., 2024). The host’s main defense system determines how TB moves from a latent to an active state. However, the MTB gene can change inside the alveolar macrophage and boosts their ability to defend against the joining of autophagosomes and lysosomes. Infections caused by MTB may manifest as either active tuberculosis disease (ATB) or asymptomatic latent tuberculosis infection (LTBI), which is influenced by the intricate biological properties of MTB and the diverse immunological responses of the host. Although most infections may be eradicated or controlled by a strong immune response, 5%–10% of infected individuals will develop ATB during their lifetime. Individuals with compromised immune systems, especially those with untreated HIV infections, are significantly more susceptible to TB compared to those with robust immune systems. The transition from latent to ATB transpires when the MTB circumvent the immune system’s defenses and commence proliferation. Some people exhibit active disease quickly after infection, whereas others manifest it later as their immune system declines (Furin et al., 2019; Aghoram et al., 2025; Ma et al., 2025; McCaffrey et al., 2022). A recent study found that single-cell RNA sequencing (scRNA-seq) increases the amount of low-abundance RNA from single cells and allows for large-scale sequencing to analyze gene activity. Also, this study examined ferroptosis-related gene activity in monocyte subsets. The TB and HC groups differed significantly, suggesting that monocyte-induced ferroptosis may contribute to TB. Non-classical monocytes expressed ferroptosis genes due to abnormal monocyte differentiation during TB development. In brief, ferroptosis protects the host from bacterial infections by suppressing intracellular pathogens through lysosomal and phagocytic processes. MTB may control ferroptosis for growth and immune evasion utilizing *FerrDBV2* (Gao et al., 2023; Ma et al., 2025). Some important genes are linked to the progression from LTBI to ATB, and a LASSO analysis found five key genes: *FBXO6, ATF3, GBP1, GBP4*, and *GBP5*. ATB patients significantly elevated the expression levels of the identified hub genes compared to LTBI patients (Chen et al., 2022). Importantly, IL-6 promotes monocyte proliferation and MTB growth after human hematopoietic stem cell infection. IL-6 activity is also associated with lung function impairment, radiological severity, and extensive TB. Elevated baseline IL-6 activity and SOCS3 induction indicate TB. Th1 cell responses, which regulate microbes, may decrease and promote immunopathology (Hamilton et al., 2025). Therefore, this review aims to analyze the various factors associated with the progression of LTBI to ATB based on the recent studies data. In this review, the distinct factors associated with the prognosis of LTBI to ATB will be analyzed through the regulation of autophagy by host-microbe interactions, the role of miRNAs in disease prognosis, single-cell RNA sequencing to examine immune responses, and the involvement of monocytes in the progression from LTBI to ATB.

## 2 Factors associated with the progression of tuberculosis

There are significant factors are links with the prognosis of the TB infection. Majorly, the MTB produce some essential genes to lose the autophagosome-lysosome fusion action. Also, other factors bring vital functions to progress the TB infection, likely, miRNAs are small, non-coded, and most of these miRNAs modulated autophagic activity or targeted ATGs to protect mycobacterial survival, contributing to TB pathogenesis. It is unclear how miRNAs alter host defenses during LTBI and if they are clinically relevant (Chen et al., 2020). Also, diagnostic advancement of single-cell sequencing utilizes to identify the genes associated with the disease progression. Furthermore, early MTB infection, monocytes, the key innate immune cells, defend the host from intracellular infections. Monocyte variety and ability to become macrophages or dendritic cells link innate and adaptive immune responses (Li et al., 2021; Wayengera et al., 2017).

### 2.1 Autophagy regulation in the progression of TB

Autophagy, a housekeeping function, maintains intracellular quality control under stress. Both innate and adaptive immunity use autophagy. New vesicles, or phagophores, grow longer and larger during autophagy and develop into the two-membrane autophagosome. The autophagosome becomes a phagolysosome for recycling or degradation after merging with the lysosome (Shariq et al., 2021). In MTB infection, autophagy has evolved from a canonical degradation pathway to a complex host-pathogen interface. Canonical autophagy, mediated by autophagy related genes (ATG) proteins like *ATG5*, promotes phagosome-lysosome fusion and reduces neutrophil-driven inflammation early in infection, limiting MTB survival in alveolar macrophages. Latest research found that total loss of autophagy components like *ATG7* and *ATG16L1* made the host more susceptible to pathogen-induced phagosome damage and macrophage necrosis (Ren et al., 2025). The MTB genome encodes eleven eukaryotic-like serine-threonine protein kinases (*STPKs*) regulate virulence, growth, metabolism, and host-pathogen interactions. *PknG* is the only *STPK* released into host cells during infection. PknG also facilitates pathogen survival during MTB infection by inhibiting macrophage phagosome maturation, which is necessary for TB pathogenicity (Ge et al., 2022). The endoplasmic reticulum (ER) stress mechanisms of MTB cause apoptosis, while autophagy helps cells survive. In contrast, the autophagy inhibition increased MTB-induced apoptosis, suggesting moderate autophagy may protect cells. In this study, *BAG2* (BCL2 associated athanogene 2) increased autophagy to reduce cell death during MTB infection, but autophagy inhibition eliminated its cytoprotective effects. *BAG2*’s role in MTB-induced ER stress and apoptosis-autophagy relationships was clarified (Liang et al., 2020). In addition, MTB infection diminished *H3K9* and *H3K27* acetylation, which is crucial for transcriptional activation, while augmenting *H3K9* and *H3K27* hypermethylation, important for transcriptional repression. These findings suggest that to enhance MTB survival, MTB PRT performs two histone modifications. Certain histone methyltransferases, such as G9a, Suv39h1/h2 (which facilitates H3K9 hypermethylation), and Ezh2 (which promotes H3K27 hypermethylation), induce histone hypermethylation (Sengupta et al., 2021). In this context, the association between *PE_PGRS* proteins and *Rab1A* enhances the *GTP activity of Rab1A*, hence boosting mTOR and preventing autophagy. Related to *RablA*-positive cells infected with the same mycobacteria, *Rab1A* knockdown cells (*siRab1A*) infected with either wild-type MTB or two complementing strains had reduced mTOR activation (measured by p-S6), suggesting that *Rab1A* regulates mTOR activity (Strong et al., 2021). Thus, the *PE_PGRS* protein of MTB suppresses the autophagy and capable of survive for g a long time in macrophages. Although, the roles of autophagy in the early detection of the transition from NIHS or LTBI to active TB illness remain largely unexplored. According to a recent study, anti-TB treatment reversed the up-regulation of *LC3B* and *ATG5* in patients with active TB and those with high bacterial burden or advanced disease. These results imply that autophagy flux could be utilized to create tools for tracking the development of TB reactivation disease (Chen et al., 2025).

### 2.2 MicroRNA association with progression of TB

**Figure 1:**
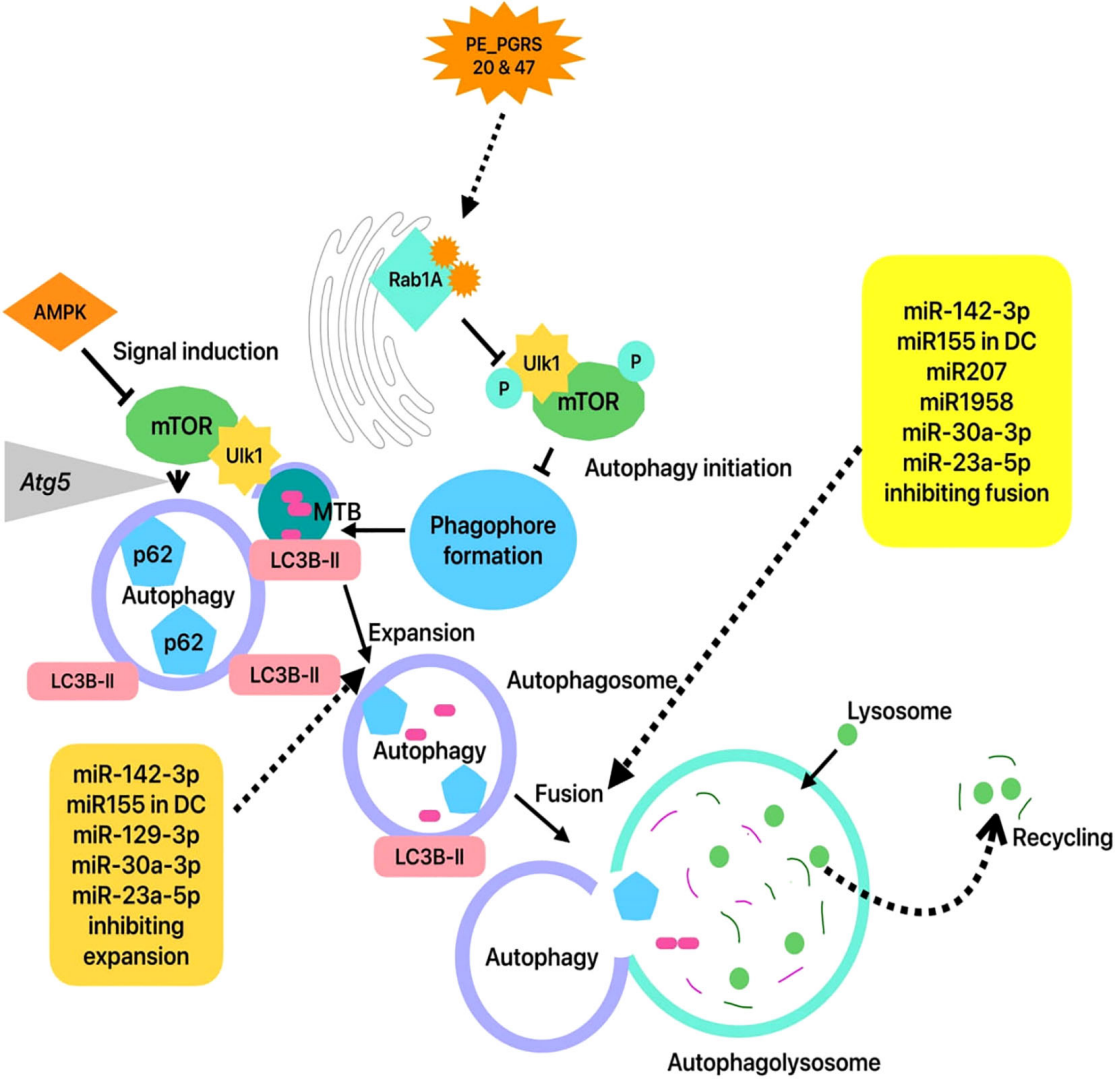
Inhibition of autophagy and role of miRNA in progression of TB. Illustrated that Autophagy in mycobacterial infection. ATGs help cells recognize nutritional or infection signals via the AMPK/mTOR pathway, activate the ULK1 complex, and recruit LC3-II to the phagophore. The phagophore, comprising LC3-II, forms an autophagosome that encloses bacteria, cytosolic organelles, and macromolecules. The autophagosome unites with the lysosome to breakdown cargo as an autophagolysosome. Thus, autophagy targeting mycobacteria involves signal induction, nucleation, growth, and fusion, which are controlled by host and bacterial stimuli. Various miRNAs inhibit the autophagy initiation, expansion, and fusion to enhance the MTB survival (Li et al., 2025). PE_PGRS proteins help MTB bacteria attach to RablA during infection. Inhibition of Ulk1 complex translocation to the preautophagosome by PE_PGRS and RablA reduces autophagy (Strong et al., 2021). DC - Dendritic cells.

MicroRNAs (miRNAs) are small, non-coding RNA molecules. By binding to the 3′-UTR regions of target mRNAs, microRNAs meticulously regulate gene expression at the post-transcriptional level. Since miRNAs have an impact on a wide range of biological functions, including immune regulation, growth, development, homeostasis, and the progression of disease. In this case, *miR-142-3p* was markedly downregulated in macrophages after MTB infection. Specifically, *miR-142-3p* negatively regulated the production of pro-inflammatory mediators (NF-κB, TNF-α, and IL-6) via targeting interleukin receptor associated kinase (*IRAK1*). The precise target of *miR-142-3p* is still ambiguous. This work revealed that miR-142-3p markedly impeded phagosome development in macrophages, mitigated autophagy caused by MTB H37Ra, and enhanced intracellular bacterial survival in macrophages by inhibiting *ATG16L1* and *ATG4c* (**Figure 1**). In contrast, *miR-106a* affects the autophagy process and mycobacterial removal in human macrophages by targeting *ULK1, ATG7*, and *ATG16L1*, which may help us better understand the host’s innate immune responses against MTB (Qu et al., 2021; Liu et al., 2021). Besides that, circRNA links to the autophagy in MTB infection, since monocytes from TB patients exhibited distinctly reduced levels of hsa_circ_0045474 compared to monocytes from healthy controls. Moreover, circRNAs have been found to participate in the autophagy of TB-associated macrophages. For instance, circRNA-0003528 facilitated MTB-associated macrophage polarization by up-regulating CTLA4 and down-regulating *miR-224-5p, miR-324-5p*, and *miR-488-5p*. Similarly, *hsa_circ_0045474 or miR-582-5p* partially mitigated the influence of *TNKS2* or *miR-582-5p* on macrophage autophagy, suggesting that the *miR-582-5p/TNKS2* axis played a role in the down-regulation of *hsa_circ_0045474* to promote macrophage autophagy in TB (**Table 1**) (Wu et al., 2022; Huang et al., 2020).

**Table 1:** Different types of genes function in the regulation of autophagy.

Study	Biological marker	Clinical outcome	Technical platform
Ren et al., 2025	CASP1, FAS, TRIM5, C5	ATB patients had significantly higher CASP1, FAS, TRIM5, and C5 mRNA expression than LTBI and HC patients.	Autophagy related genes (ARG) signature
Ge et al., 2022	PknG/ LC3-ll puncta/ SQSTM1	Mtb PknG inhibit autophagosome function with kinase activity. Infected cells with WT Mtb, ΔpknG:pknG, or ΔpknG:pknGK181M strains showed elevated LC3-II and SQSTM1 protein levels.	Western blot
Liang et al., 2020	BAG2	Selective BAG2-induced autophagosomes combine with lysosomes. The vacuolar H+-ATPase inhibitor bafilomycin A1 (Baf A1) increased autophagic vesicle marker LC3-II, while Bag2 siRNA inhibited this accumulation.	MTB H37Ra-infected BMDMs and RAW264.7 cells, assess autophagosome formation kinetics using western blotting.
Qu et al., 2021	miR-142-3p	Overexpressing miR-142-3p reduced H37Ra-induced autophagy activation, prevented macrophage phagosome formation, and increased MTB macrophage survival.	MTB infected in RAW264.7 cells
Liu et al., 2021	miR-106a	Induced miR-106a expression improved intracellular mycobacterial survival, but transfection with miR-106a inhibitors lowered it.	TEM

miR, microRNA; TEM, Transmission Electron Microscope; BMDM, Bone marrow derived macrophages;mRNA, messenger Ribonucleic acid; BAG2, BCL2 associated athanogene 2; SQSTM1, sequestosome 1; pknG, protein kinase G; LC3, Microtubule, associated protein 1A/1B light chain 3.

Extracellular vesicles (EVs) and exosomes, which transport proteins, mRNAs, and non-coding RNAs (ncRNAs), are essential for facilitating intercellular communication among proximate and remote cells, thereby influencing numerous cellular and biological processes. Noticeably, the plasma exosomes from LTBI patients transmitted *miR-7850-5p* to THP1, inhibiting the expression of the critical iron death gene *SLC11A1* and promoting MTB’s intracellular survival in THP1 (Cui et al., 2024; Shahzad et al., 2022). In addition, *miR-144-3p* has the potential to inhibit the activation of autophagy and enhance BCG infection through its targeting of ATG4a in RAW264.7 cells. In macrophage cells, *miR-23a-5p* may impact autophagy and mycobacterial viability by targeting TLR2 within the TLR2/MyD88/NF-κB signaling pathway during MTB infection (Lyu et al., 2019).

### 2.3 Single-cell RNA sequencing in host immune response

Single-cell lung events can cause or control TB. Single-cell technologies like RNA-sequencing (scRNA-seq) can objectively profile immune cell populations in healthy and diseased humans and animals. Although circulating NK cells are highly important in TB immunity, besides the study found CD27+ NK cells in macaque hosts’ lungs that regulate MTB infection. Memory-like responses and vaccine-induced MTB protection depend on CD27+ NK cell recognition, proliferation, and survival. The TNF receptor superfamily member CD27 may indicate mature or memory natural killer cells. In cytotoxicity, cytokine production, proliferation, and function, CD27+ NK cells outperform CD27-NK cells. Also, this study findings support NK cell TB protection. In LTBI patients, mycobacteria increase CD27+ NK cell growth. Human monocytes and AMs infected with MTB can be lysed by NK cells to boost immunity (Esaulova et al., 2021; Venkatasubramanian et al., 2017). Also, a similar study scRNA-seq analyzed distribution of γδT cell sub-clusters across the tested groups indicated that latent MTB infection induces a specific proliferation of CD81bright γδ T cells, skewing them towards AKT signaling, which is mediated by PI3K and involves cell activation through the second messenger PIP3. The PI3K/AKT pathway is a crucial signaling cascade that regulates metabolism, cellular growth, and survival (Shekarkar Azgomi et al., 2024). Furthermore, in CyTOF analysis reveals that non-progressing LTBI patients have fewer MAIT, NKT, and NK cells than those with PTB, suggesting lower microbial abundance. These cells are essential for mycobacteria regulation. PBMCs of LTBI patients who developed PTB had more CD16+CD56dimCD57+ early NK cells. Antigen-dependent cell cytotoxicity (ADCC) indicates microbicidal activity via CD16 (Fc gamma receptor III), while reduced CD56 and elevated CD57 indicate cellular senescence. Compared to active pulmonary TB (PTB), latent TB infection progression may have more CD16+CD56dimCD57+ early natural killer (NK) cells, suggesting the involvement of antibody-dependent cellular cytotoxicity (ADCC) cells that regulate organisms. In addition, the MAIT NKT cells contribute significantly to the initial response to mycobacteria and are effectively equipped with cytotoxic molecules through granzyme B activity (Kamolratanakul et al., 2024; Ruibal et al., 2021). In a recent study indicates that these distinct genes are essential for TB that gp91-phox subunit of the phagocytic oxidase complex, responsible for producing superoxide and other reactive oxygen species necessary for microbial destruction, is encoded by the *CYBB* gene. *CYBB* overexpression elevates reactive oxygen species (ROS), which can damage tissue and initiate clinical symptoms associated with LTBI. The ROS have historically been considered responsible for tissue damage in both acute and chronic inflammatory diseases. Notably, this study employed machine learning algorithms Lasso, random forest (RF), and SVM-RFE to identify specific genes associated with TB development and the inflammatory response (Ma et al., 2023; Frazão et al., 2015). In brief, to comprehend gp91phox, historically referred to as cytochrome b558, reveals that the NOX2 core complex comprises two transmembrane subunits: the auxiliary p22phox (p22) subunit and the catalytic NOX2 subunit, also known as gp91phox (Noreng et al., 2022). Recent findings from flow cytometry and single-cell RNA sequencing indicate an increase in the fraction of monocytes associated with active TB. However, the pathophysiology of TB, mortality was associated with monocyte dysfunction and the activation of the innate immune system (Janssen et al., 2017).

### 2.4 Monocytes associated with LTBI to ATB

The fatal illness TB is associated with monocytes. Though, numerous studies have demonstrated that autoimmunity has a role in TB. The innate and adaptive immune systems are essential for combating MTB. Cells of the innate and adaptive immune responses are involved in TB granuloma, with a particular emphasis on monocytes. In a recent study, the PPI network contained three essential monocyte-related genes *SERPINA1, FUCA2*, and *HP*. *Haptoglobin (HP)* is the only gene directly connected to TB development and activity. In an acute-phase response, the haptoglobin protein aids tissue repair, infection prevention, and internal environment stability. In TB pleurisy, HP expression is high (Li et al., 2021; Gao et al., 2019). The study’s clinical sample verification found that active TB patients’ PBMCs had higher HP levels than latent TB and healthy controls. This study suggested that tissue hypoxia raises HP levels in active TB. Under hypoxia, many red blood cells lysed, raising tissue hemoglobin levels. As hemoglobin is absorbed, haptoglobin expression rises. Patients with active TB were sensitive. Serine protease inhibitor A1 (SERPINA1) inhibits over 90% of plasma proteases due to its highly conserved protein structure. Arya et al. found that active TB had high *SERPINA1* expression using the *iTRAQ-I* experiment and Western blotting. Acute-phase reactive protein is mostly produced by liver cells, however epithelial, monocyte, and macrophages can also manufacture it. Active TB also causes lung inflammation (Li et al., 2021; Arya et al., 2020; Zoller et al., 2018). Notably, the PTB patients displayed a diminished proportion of CD14+CD16− monocytes and an increased prevalence of CD14+CD16+ and CD14−CD16+ monocytes in comparison to healthy controls. Monocytes contain CD163, a scavenger receptor that endocytoses haptoglobin-hemoglobin complexes and is indicative of M2 macrophages (Canton et al., 2013). The proliferation of CD16+CD163+MerTK+ monocytes in TB diminishes the host’s defense against MTB due to their reduced pro-/anti-inflammatory cytokine production ratio and restricted T cell activation. CD163 and MerTK signify M2-like macrophage activation, characterized by immunomodulatory and pathogen-permissive properties (Liu et al., 2019; Lastrucci et al., 2015). To comprehend the *DosR* (dormancy of survival) regulon in the LTBI state, a segment of the MTB genome activated during dormancy comprises 50 genes. Patients with LTBI exhibit more pronounced immune responses to these antigens compared to those with active TB, hence facilitating the differentiation between infection and disease (Meier et al., 2018). These antigens may help assess ATB treatment success as well as distinguish LTBI from ATB. The most promising M. tuberculosis *DosR* regulon encoded antigens were *Rv0081, Rv1733c, Rv1737c, Rv2029c*, and *Rv2628*, which showed immunogenic potential across studies and regions. The *DosR* regulon’s transcriptional regulator *Rv0081* may be important in hypoxia. Long-term incubation studies in Ethiopia and South Africa showed this antigen’s immunogenicity (Mensah et al., 2014; Araujo et al., 2015; Bai et al., 2016). The median monocyte-to-lymphocyte ratio (MLR) in IGRA+ subgroup B was 0.52, greater than subgroup A (0.35, p = 0.04). This ratio is linked to blood transcriptomes and TB risk. However, the IGRA+ B subgroup, MLR level increased marginally (Broderick et al., 2021; Naranbhai et al., 2015).

### 2.5 Future perspectives on targeting biomarkers on TB prognosis

Multiple factors contribute to the progression of TB, particularly as MTB employs various genes for intracellular survival. Analyses of the transcriptome and proteome facilitate the development of diagnostic markers and antimycobacterial agents. Small RNAs, including miRNA, lncRNA, circRNA, and the ceRNA network, will have important roles in gene regulation and translational research. In this context, numerous miRNAs are essential in the regulation of autophagy; specifically, *miR-155* inhibits autophagy in human dendritic cells by targeting *ATG3*. In macrophages infected with MTB, *miR-17-5p* regulates autophagy by targeting *Mcl-1* and *STAT3*. *MiR-27a* facilitates the survival of MTB within cells by modulating Ca2+-associated autophagy. In human monocytes and macrophages, *miR-144-5p* suppresses antibacterial autophagy and the innate immune response to MTB by targeting *DRAM2*. MTB inhibits integrated autophagic pathways that promote bacterial intracellular survival and persistence through the production of *miR-33* and miR-33*. Research indicates that *miR-20a* targets *ATG7* and *ATG16L1*, resulting in reduced autophagy and increased BCG survival in murine macrophages (Etna et al., 2018; Liu et al., 2018; Kim et al., 2017; Liu et al., 2021). Thus, analyses of small RNAs and their interaction with autophagy in clinical strains will identify potential diagnostic markers. Pathogenic mycobacteria expressing PE and PPE families inhibit autophagy, thereby enhancing bacterial survival within macrophages during host-microbe interactions. The expression of PE or PPE proteins markedly enhanced mTOR signaling in *Mycobacterium smegmatis*, indicating a potential mechanism for the inhibition of autophagy induced by mycobacteria (Strong et al., 2020). This analysis of mycobacterial self-defense mechanisms against autophagosome-lysosome fusion presents a promising tool for the development of antimycobacterial agents.

## 3 Discussion

This review provides a comprehensive analysis of the factors associated with the progression of LTBI to ATB. Majorly, various types of genes significantly modulate the immune responses of MTB and host interactions (**Table 2**). Particularly, analyses of autophagy-regulated genes and their association with MTB survival are crucial to determine the disease prognosis, a vital cellular response to mycobacterial infection, has garnered significant attention. The PE and PPE proteins identified as regulators of autophagy in infected cells comprise *PE_PGRS47, PE_PGRS41, MMAR_0242*, and *PE_PGRS29*. This protein’s role in inhibiting autophagy in MTB-infected cells is highlighted by the identification of a Tn insertion mutation in the gene encoding *PE_PGRS47* (Strong et al., 2020; Saini et al., 2016; Deng et al., 2017; Chai et al., 2019). In addition, *SIRT4*, a member of the sirtuin family, is located in the mitochondria. The functions encompass substrate-specific deacetylase, lipoamidase, ADP-ribosyltransferase, and deacetylase activities. *SIRT4* is also associated with cellular defense mechanisms against microorganisms. In cells treated with LPS, the overexpression of *SIRT4* enhanced steroidogenesis and reduced apoptosis, thereby promoting the dissemination of MTB infection and the activation of LTBI (Min et al., 2019; Ramatchandirin et al., 2016). The “risk of progression to ATB” signature exhibited a significant correlation with the LTBI-Risk treatment signature. Four predictive gene profiles (*BATF2, RISK6, Zak16*, and *Sweeney3*) indicated that LTBI-Risk patients exhibiting elevated expression of genes linked to the “risk of progression to ATB” post anti-TB treatment demonstrated significant downregulation of numerous genes. In contrast to therapy-unaffected genes, these genes exhibited enrichment for IFN signaling and functional protein-protein interactions (Burel et al., 2021; Zak et al., 2016; Sweeney et al., 2016; Warsinske et al., 2018; Roe et al., 2020; Penn-Nicholson et al., 2020).

**Table 2:** Clinical immune responses in progression of TB infection.

Study	Biological marker	Clinical outcome	Study population/ Technical platform
Venkatasubramanian et al., 2017	IL-21	CD3-CD56+CD27+ cells inhibit M. tb H37Rv growth in macrophages more than CD3-CD56+CD27- cells. Unlike scrambled siRNA, IL-21 siRNA inhibited ESAT6-dependent NKp46+CD27+KLRG1+ cell growth.	12 individuals in positive QuantiFERON-TB Gold tests- (+)LTBI, while 12 others had negative tests- HC.
Shekarkar Azgomi et al., 2024	CD81+bright γδ+	TBI samples have increased IL2RB, ICOS, LTB, GPX4, and CD79B. However, SYT11, CD81, XBP1, and GPX4 changed synchronously in the IL-2-STAT5 signaling pathway. TBI also leads to an increase of CD81+bright γδ+ T cell subsets.	Total-15 participants were enrolled: ATB-5, with TBI-5, and HD-5.
Kamolratanakul et al., 2024	MAIT NKT	People with latent tuberculosis infection (LTBI) had more MAIT NKT cells than those with active PTB and healthy controls. In addition, 6 of 17 (35%) LTBI patients advanced to active PTB and had more MAIT NKT cells and early NKT cells than those without progression.	A total of 32 subjects (12 PTB, 17 LTBI, 3 healthy volunteers)
Liu et al., 2019	CD14+CD16+	Patients with PTB had higher rates of CD14+CD16+ monocytes (15.7% vs 7.8%, P < 0.0001), CD14-CD16+ monocytes (5.3% vs 2.5%, P = 0.0011), and lower percentage of CD14+CD16-cells (51.0% vs 70.4%, P = 0.0110) compared to healthy controls (HC	129 people included, LTBI= 20, malignant pleural effusion (MPE) = 21), PTB= 39), tuberculous pleurisy = 28), healthy controls = 21.
Ding et al., 2025	CD4+/CD8+ T-cell	The intermediate CD4+/CD8+ T-cell ratio in LTBI cases indicated dynamic immunological balance. The tuberculosis cohort had more inflammatory T-cells and CD8+ T-cell-mediated MHC-I and BTLA signaling.	Single-cell RNA sequencing (scRNA-seq) -evaluate PBMCs from 7 people: 2 HC, 2-LTBI patients, and 3-active tuberculosis (ATB) patients.
Shariq et al., 2021	*RipA*	In response to *RipA*, macrophages produce pro-inflammatory cytokines TNF-α, IL-6, and IL-12 in a dose- and time-dependent manner. Additionally, TLR4 recognizing RipA can lead to macrophages releasing IL-6, IL-12, and TNF-α.	In-silico
Strong et al., 2021	*PE_PGRS20 & 47, Rab1A*	*Rab1A* with *PE_PGRS20* or *PE_PGRS47* reduced proinflammatory cytokine production and MHC class II-restricted antigen presentation.	immunoblots/ Infection of RAW 264.7 macrophages

MTB, Mycobacterium tuberculosis; H37Rv, Human 37 Rough virulent; siRNA, small interfering RNA; ESAT6, Early Secreted Antigenic Target 6; LTBI, Latent TB infection; PTB, Pulmonary TB; TLR, Toll like Receptor; IL, Interleukin; PE_PGRS, polymorphic GC, rich repetitive sequence; TNF, Tumour necrosis factor; MHC, 1, Major Histocompatibility Complex, 1; CD14 &16, Cluster of Differentiation 14&16; MAIT NKT cells, Natural Killer T (NKT) and Mucosal, Associated Invariant T (MAIT) cells.

Multiple studies show that TB increases complement component gene expression. TB genes had higher *C1q*-encoding gene expression than LTBI and other lung illnesses. However, whole blood RNA studies reveal monocytes or macrophages promote *C1q* gene transcription (Lubbers et al., 2018; Scriba et al., 2017; Jiang et al., 2017; Blankley et al., 2016; Petrilli et al., 2020). The transcriptome study of differentially expressed genes (DEGs) associated in TB development showed that *miR-155* targeted Rheb to increase macrophage autophagy to eliminate intracellular Mycobacteria. However, *miR-155* targets *ATG3* to inhibit human dendritic cell autophagy. *MiR-17-5p* targets *Mcl-1* and *STAT3* to control autophagy in MTB-infected macrophages (Liu et al., 2021). So DEGs analysis is critical for ATB and LTBI. Also, *SULT1A3*, connected to treatment success in adults with TB, and *NCOA3*, which showed differential miRNA expression between hospitalized TB patients and controls. *DEFA1* and *DEFA3*, biomarkers for differentiating ATB and LTBI in children, predicted TST conversion better (Bobak et al., 2023; Esaulova et al., 2021; Silva et al., 2021).

The WHO recommends various diagnostic approaches to identify high-risk populations and expedite the detection and treatment of TB (Zhang et al., 2025). Whole-genome microarrays and, to a lesser extent, RNA-seq have shown that analyzing gene activity can elucidate the mechanisms of infection spread (Estévez et al., 2020). However, the recent study supports a substantial correlation between disease progression and transcriptome-associated genes that aid in the survival of intracellular bacilli in macrophages (*ORL1*). Also, genes that code for two carriers of cobalamin (vitamin B12), a metabolite that might be involved in MTB pathogenesis. ATB patients may have better vitamin B12 absorption and bacterial survival if their expression of those carriers is higher. In addition, another study revealed that the crucial role of transcriptome data was supported by the upregulation of syndecans (*SDC1, SDC3*, and *SDC4*), the complement cascade, and type I and II interferon signaling in TB patients (Singhania et al., 2018; Sambarey et al., 2017).

On the other hand, host immune response and MTB survival must be examined. CCRL2 is engaged in C-C chemokine ligand type 2 signaling, MAPK14 is a mitogen-activated protein kinase 14, and MSR1 is a receptor involved in TB progression/phagocytosis and destruction. B, CD4+, and CD8+ T cells are adaptive immune cells. Depletion of CD4+ T cells, like HIV infection, increases TB risk (Nangpal et al., 2025). A subsequent transcriptome study of LTBI’s hematological immune-mediated features confirmed the findings. LTBI patients had more active memory CD4+ and CD8+ T cells, cytolytic activity, and T-cell co-inhibition. This supports recent findings that persistent infections require enhanced cytolytic activity and regulatory mechanisms to maintain immunological homeostasis (Liu et al., 2025; Bolivar-Wagers et al., 2022; Teh et al., 2022), IL-18 is essential for IFN-γ production, hence decreased levels indicate a less adaptive immune response. Reduced IL-18 levels enhance MTB growth since interferon-gamma is related with the T-helper type 1 profile, which fights this infection. TNF-α expression is crucial for preserving tuberculous granulomas and avoiding LTBI reactivation (de Oliveira Rezende et al., 2022; Buha et al., 2019; Silva et al., 2018). The CD38+CD27 pattern of CD4 T cells distinguished active pulmonary tuberculosis (PTB) from non-tuberculosis (Yang et al., 2025). Disease development depends on MTB culture filtrate protein-10 (CFP-10), early secreted antigenic target-6 (ESAT-6), and purified protein derivative (PPD) antigenicity. PPD activation may increase transcriptional gene profile differentiation, prompting a larger range of host immune responses (Wang et al., 2019; Wu et al., 2017; Clifford et al., 2019).

Key genetic components of protective immunity in human TB include TNF-A, IL-1B, IFN-γ, and TLR-2. TLR4 rs4986791, TLR2 rs5743708, TNFA rs361525, IL1B rs1143627, and IFNG rs2430561 are immune-related SNPs associated with tuberculosis susceptibility (Cubillos-Angulo et al., 2019; Zhou et al., 2017; Wei et al., 2017; Amaral et al., 2018). Active TB and LTB patients have much greater IL-18 and IL-18BP mRNA levels than healthy controls. This may indicate that immune cell signaling pathways are constantly stimulated during MTB infection to control or promote active TB. IL-18BP, an endogenous inhibitor, controls IL-18 levels. Normal plasma IL-18BP is 20 times higher than IL-18, preventing IL-18 from attaching to its cellular receptor. Chromosome 11 contains the IL-18BP gene at 11q13.4. Two response elements in the mRNA promoter region control gene expression and protein synthesis after IFN-γ attachment. Thus, biological indicators are molecular diagnostics for LTBI and ATB (Wawrocki et al., 2020; Hong et al., 2022; Ding et al., 2025).

In *DosR* regulon, the dominant T cell antigen and transmembrane protein *Rv1733c* is revealed via bioinformatics. This antigen triggers higher immune responses in LTBI patients than ATB and healthy controls. In the top 45 of 189 antigens was *Rv2628*. Its purpose is unclear. Many studies found that LTBI patients had better immune responses than ATB patients. Additionally, *Rv2031c* (α-crystallin or heat shock protein X) is a crucial *DosR* regulon antigen for MTB growth in macrophages during latency. *Rv1737c* may carry nitrate. Four long-term immunogenicity tests showed LTBI patients outperformed ATB patients. *Rv2029c*, a glycolysis-related phosphofructokinase, has been studied in mice as a vaccine candidate (Su et al., 2017; Arroyo et al., 2016; Meier et al., 2018). In addition, the ML ratio captures monocyte qualitative changes better than monocyte counts, which may explain its epidemiologic linkages. The recent research and enrichment of interferon signaling imply that type I and type II interferons vary the ML ratio and monocyte function enough to explain changed disease history, consistent with their major involvement in mycobacterial and inflammatory disorders (Naranbhai et al., 2015; Mayer-Barber et al., 2014).

## 4 Conclusion

This review analyzes the recent research on factors linked to the progression from latent to active TB. Mycobacterial genes that regulate autophagy play a critical role in disease prognosis. The T-cell-mediated immune response and transcriptome functions in TB prognosis enable the differentiation between active and latent tuberculosis, improve disease prognosis, facilitate the identification of diagnostic biomarkers, and address the existing research gap.
