# Correction: Functional analysis of distinct factors linked to the development of latent to active tuberculosis

**DOI:** 10.3389/fcimb.2026.1817346

**Published:** 2026-03-10

**Authors:** Karthikeyan Sundaram, Venkataraman Prabhu

**Affiliations:** 1 Department of Herbal Pharmacology and Environmental Sustainability, Chettinad Hospital and Research Institute, Chettinad Academy of Research and Education, Kelambakkam, Chennai, Tamilnadu, India; 2 Division of Medical Research, SRM Medical College Hospital and Research Centre, Kattankulathur, Chennai, Tamilnadu, India

**Keywords:** tuberculosis, active tuberculosis, latent tuberculosis, autophagy, immune response

In the published article, there was a mistake in the **Funding** statement. The correct statement is: “The authors gratefully acknowledge the financial support provided by SRM Medical College Hospital and Research Centre, Faculty of Medicine and Health Sciences, SRMIST, Kattankulathur, Tamil Nadu, India.”

The original version of this article has been updated.

